# Thyroid Dysfunction as a Predictive Indicator in Camrelizumab of Advanced Esophageal Squamous Cell Carcinoma

**DOI:** 10.1155/2022/4015897

**Published:** 2022-07-04

**Authors:** Ying Chen, Lei Zhuang, Danhong Zhang, Xianghui Du, Liming Sheng

**Affiliations:** ^1^Department of Thoracic Radiotherapy, The Cancer Hospital of the University of Chinese Academy of Sciences (Zhejiang Cancer Hospital), Hangzhou, Zhejiang 310022, China; ^2^Institute of Basic Medicine and Cancer (IBMC), Chinese Academy of Sciences, Hangzhou, Zhejiang 310022, China; ^3^Key Laboratory Diagnosis & Treatment Technology on Thoracic Oncology, Hangzhou, Zhejiang 310022, China; ^4^The Second Clinical Medical College, Zhejiang Chinese Medical University, Hangzhou, Zhejiang 310022, China

## Abstract

Thyroid dysfunction (TD) induced by programmed death-1 (PD-1) or programmed cell death-ligand 1 (PD-L1) immune checkpoint inhibitors (ICIs) has been widely reported. However, the effects of ICI-induced TD on the survival of patients with esophageal squamous cell carcinoma (ESCC) have not been described. Herein, a retrospective study was conducted, which 82 patients with advanced metastatic or recurrent ESCC treated with camrelizumab were enrolled. Twenty patients (24.4%) experienced TD during camrelizumab treatment with or without chemotherapy. The median onset time of TD was 1.7 months. The incidence of TD was 35.6% in patients who previously received thoracic radiotherapy versus 10.8% in patients who did not (P =0.009). Patients with TD had significantly longer median progression-free survival (5.5 months vs 3.5 months, P =0.035) and overall survival (26.7 months vs 11.5 months, P <0.001). TD is frequently observed in ESCC patients treated with camrelizumab and especially in patients who received radiotherapy previously. ESCC patients with TD during ICIs treatment often have better prognosis.

## 1. Introduction

Esophageal cancer is one of the malignant tumors with terrible prognosis. The substantial majority of esophageal cancer patients are in East Asia, and most patients suffered from squamous cell carcinoma [1]. Patients with metastatic or recurrent esophageal squamous cell carcinoma (ESCC) have very poor prognosis due to insensitivity to chemotherapy [2]. In the past decade, immunotherapy has become the most promising progress in solid tumor treatment. Typically, in the field of lung cancer [3] and melanoma [4], programmed death-1 (PD-1) and programmed cell death-ligand 1 (PD-L1) inhibitors greatly prolongs the survival time of such patients. As a revolutionary treatment, immunotherapy has also been slowly used to treat ESCC [5]. The discrepancy in pathological types of esophageal cancer between Eastern countries and Western countries lead to huge differences in certain genes mutation frequencies [6]. This also indirectly leads to the possibility that immunotherapy may have different clinical profiles in patients with ESCC [6].

Cytotoxic T lymphocyte antigen 4 (CTLA-4) or PD-1 and its ligand PD-L1 are the two most important targets of immune checkpoint inhibitors (ICIs). By inhibiting them, ICIs could activate T cells and thereby play a key role in killing tumor cells. The representative drugs include nivolumab, pembrolizumab, durvalumab, and ipilimumab, which have been widely used in numerous cancer treatment. In recent years, it has been found that compared with traditional chemotherapy, ICIs, like nivolumab [7], pembrolizumab [8], and camrelizumab [9], could greatly prolong the overall survival (OS) of patients with advanced ESCC. Moreover, patients treated with ICIs suffered from less treatment-related side effects and had better quality of life. Camrelizumab is a highly selective humanized PD-1 monoclonal antibody developed in China. It showed durable tumor response and improvement in OS with comparably less toxicity in metastatic ESCC [9]. Combined use of camrelizumab on the basis of chemotherapy can achieve higher tumor remission and longer OS in untreated advanced ESCC [10]. Therefore, camrelizumab has become a new treatment option and standard for advanced ESCC patients in China.

While the therapeutic efficacy is improved, patients treated with ICIs still experience the immunotherapy-related adverse events (irAEs). irAEs are fundamentally different from those of traditional anti-tumor therapy [11]. Prompt identification and treatment of irAEs could help patients obtain the maximum benefit from this promising therapy. Interestingly, the arise and severity of irAEs are closed related to therapeutic effect and prognosis. Generally, patients with irAEs had superior tumor response and longer OS [12–14]. However, some reports demonstrated that irAEs are not related to prognosis in cancer patients who received ICIs [15].

The thyroid is one of the most frequent target organs affected by PD-1 or PD-L1 inhibitors. Hyperthyroidism and hypothyroidism are the two most common thyroid dysfunctions (TDs) in patients treated with ICIs. Generally, TD often manifests as an early onset of transient thyrotoxicosis, followed by hypothyroidism [16]. Several studies reported that 10-20% of lung cancer patients would have TD during ICIs treatment. Cancer-specific survival was often better in patients with TD than that without TD [17, 18]. However, there are few reports on the incidence of TD in ESCC patients and it is also very urgent to know the relationship between TD and the effect of ICIs. Therefore, this retrospective study was designed to investigate the occurence and risk factors of TD induced by camrelizumab and their relationship to outcome in ESCC patients.

## 2. Methods

### 2.1. Patients

A retrospective study involving patients with advanced metastatic or recurrent ESCC was conducted. ESCC patients treated with camrelizumab between January 2019 and August 2021 at the Cancer Hospital of the University of Chinese Academy of Sciences (Zhejiang Cancer hospital) were enrolled in this study. The inclusion criteria were as follows: advanced metastatic or recurrent ESCC confirmed by histology or cytology; age between 18 years and 75 years; received at least two cycles of camrelizumab with or without chemotherapy; no previous thyroid disease, including thyroiditis, hyperthyroidism, hypothyroidism, Graves' disease, and thyroid cancer; regular blood thyroid function test during immunotherapy. The exclusion criteria were as follows: second primary cancer in addition to esophageal cancer in the past or currently; abnormal thyroid function within one week before camrelizumab treatment; previously underwent thyroidectomy; previously prescribed with either levothyroxine or methimazole. Patients' clinicopathological characteristics, such as age, gender, performance status (PS) score, smoking status, tumor location, TNM staging, and previous treatment, were collected from medical records. This study was approved by the institutional review board of The Cancer Hospital of the University of Chinese Academy of Sciences (Zhejiang Cancer hospital).

### 2.2. Camrelizumab treatment

All patients received camrelizumab three-week cycle with or without chemotherapy. It was given at a fixed dose of 200 mg/cycle intravenously over approximately 30 minutes. If it was used in combination with chemotherapy, the chemotherapy agents should be used after camrelizumab injection. Camrelizumab would be used for at least two years only stopped in case of disease progression, intolerable adverse events, and patients' decision. Once immune-related adverse events occurred, camrelizumab could be interrupted for up to six weeks.

### 2.3. Thyroid function tests and classification of TD

Thyroid function tests were carried out in our hospital's clinical laboratory using electrochemiluminescent bridging immunoassay. Blood samples were drawn and tested for free thyroxin (fT4), free triiodothyronine (fT3) and thyroid-stimulating hormone (TSH). Thyroid function tests were performed seven days within the initiation of camrelizumab, as well as every cycle of immunotherapy.

The reference ranges of fT4 and TSH in our hospital were 0.81–1.89 ng/dL and 0.38–4.34 *μ*IU/ml, respectively. Two physicians independently reviewed the thyroid function results. On the basis of fT4 and TSH values, TD was divided into two groups: hypothyroidism and hyperthyroidism. When fT4 was within the normal range and TSH was low (0.38 < *μ*IU/ml) or high (>4.34 *μ*IU/ml), TD was scored as “subclinical hyperthyroidism” or “subclinical hypothyroidism,”, respectively. When TSH was low (0.38 < *μ*IU/ml) and fT4 was high (>1.89 ng/dL), TD was scored as “overt hyperthyroidism,” or *vice versa* (overt hypothyroidism). TThe Common Terminology Criteria for Adverse Events version 5.0 (CTCAE v5.0.) was utilized to grade TD toxicities. The time of appearance of TD was defined as the time interval from the first use of camrelizumab to the first time TD was diagnosed.

### 2.4. Tumor response evaluation and outcomes

Every patient underwent computed tomography scans of the chest and upper abdomen, and supraclavicular B-ultrasound every six weeks. Response Evaluation Criteria in Solid Tumors (RECIST) was utilized to assess tumor response. The primary endpoints in this study were progression-free survival (PFS) and OS. The time from the first injection of camrelizumab to cancer progression or death was calculated as PFS. OS was defined as the interval between the first injection of camrelizumab and death from any cause or censored at the date of last follow-up.

### 2.5. Statistical analysis

The categorical variables were evaluated by Chi-square test. Fisher's exact test was carried out when Chi-square test assumptions were not valid. Survival curves were estimated by the univariate Kaplan–Meier method. The median survival time with corresponding 95% confidence intervals (CIs) were presented. To investigate the influence of risk factors on survival, hazard rations (HRs) and 95% CIs were calculated using univariate Cox proportional hazards models. Cox proportional hazards models were fitted to adjust the effect of TD for potentially prognostic covariates, such as patients' gender, age, performance score (PS), treatment lines, tumor stage, and liver metastasis. All statistical analyses were carried out with SPSS 26.0 for Windows. Statistical significance was considered at P <0.05.

## 3. Results

### 3.1. Patients

A total of 82 patients with advanced metastatic or recurrent ESCC treated with camrelizumab were enrolled in this study. The vast majority of patients were men (76/82, 92.7%). The ratio of men to women was 12.7 : 1. The median age of the entire group of patients was 63 years. Twenty-three patients received camrelizumab monotherapy, whereas 59 patients received immunotherapy combined with chemotherapy. Most patients (62/82, 75.6%) had a history of smoking. There were 11, 42, and 29 cases of esophageal cancer in the neck and upper chest, middle chest, and lower chest, respectively. First-line treatment with camrelizumab was administered to 25 patients for a median of six cycles, whereas the remaining 57 received second-line or further treatment for a median of four cycles. The detailed characteristics of patients are shown in Table 1.

### 3.2. Association between clinicopathological variables and TD

Twenty patients (24.4%) experienced TD during camrelizumab treatment with or without chemotherapy. The most common TD subtype was subclinical hypothyroidism (11 patients, 55.0%), followed by overt hypothyroidism (5 patients, 25.0%), subclinical hyperthyroidism (2 patients, 10.0%), and overt hyperthyroidism (2 patients, 10.0%). According to CTCAE v5.0, only one patient was graded as 3, and this patient continued to receive immunotherapy after levothyroxine replacement. The vast majority of patients were graded as 1 or 2. The median onset time of TD was 1.7 months (range: 0.3–13.7 months). The median onset time of hypothyroidism was shorter than that of hyperthyroidism (1.7 months vs 2.4 months, P <0.05).

We performed Chi-square test to investigate the association between clinicopathological variables and TD (Shown in Table 1). Patients without liver metastasis were prone to have TD, but the difference was not statistically significant (P =0.051). The incidence of TD was 35.6% in patients who previously received thoracic radiotherapy versus 10.8% in patients who did not (P =0.009). Additionally, patients' PS score was also related to TD (P =0.032). Patients with PS = 0 tended to develop TD during immunotherapy. There is no relationship between the site of radiation therapy and radiation therapy-induced hypothyroidism, calculated by chi-square test (P = 0.502). Moreover, TD was not associated with gender, age, smoking, alcohol, treatment lines, and tumor stage (P > 0.05).

### 3.3. Clinical response and survival analysis

Thirteen patients (15.9%) were confirmed to have PR, 63 patients SD, and 6 patients PD. PR was positively correlated with the incidence rates of TD (P = 0.002).

**Figure 1:**
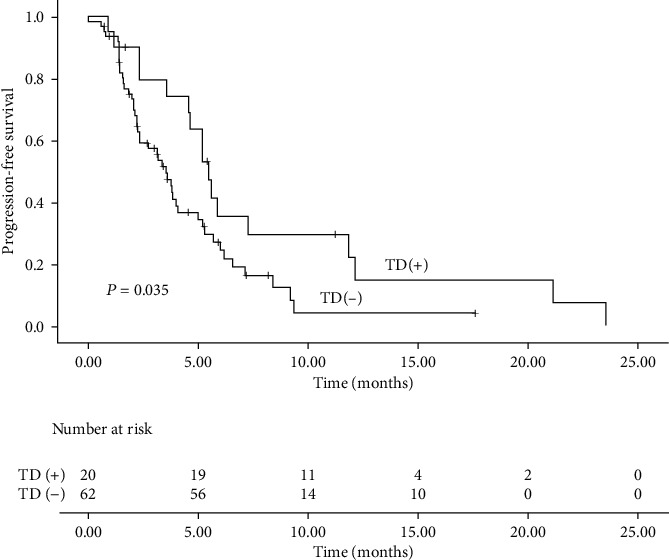
Progression-free survival in ESCC patients with and without TD.

**Figure 2:**
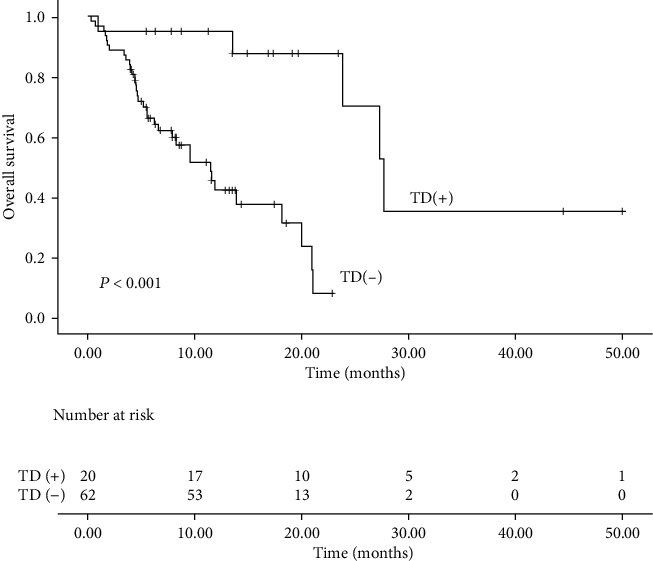
Overall survival in ESCC patients with and without TD.

**Figure 3:**
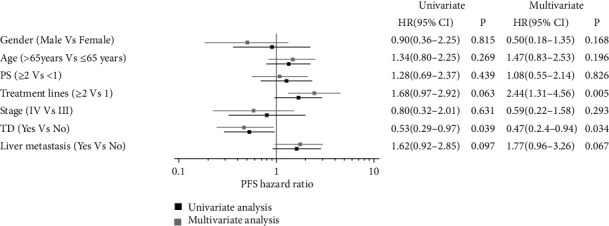
Prognostic factors for PFS by univariate and multivariate analysis in ESCC patients received camrelizumab.

**Figure 4:**
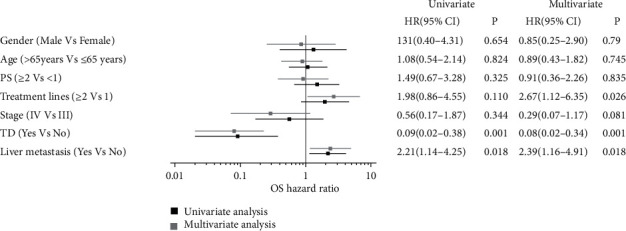
Prognostic factors for OS by univariate and multivariate analysis in ESCC patients received camrelizumab.

The median follow-up time was 15.3 months (range: 3–50 months). Fifty-seven patients discontinued camrelizumab due to cancer progression. Nine patients experienced intolerable irAEs. The most common serious side effects that caused the cessation of camrelizumab were esophageal perforation (3 patients, 3.7%) and pneumonia (3 patients, 3.7%). No patient discontinued immunotherapy due to TD. The median PFS and OS were 3.8 and 18.2 months, respectively. Sixty-three patients (76.8%) progressed during immunotherapy, and 39 patients (47.6%) died. Patients with TD had significantly longer median PFS (5.5 months vs 3.5 months, P =0.035, Figure 1) and OS (26.7 months vs 11.5 months, P <0.001, Figure 2) compared with patients without TD. Multivariate Cox regression analysis for PFS (Figure 3) showed that TD and treatment lines were significantly associated with PFS (TD: HR = 0.47, 95% CI = 0.24–0.94, P = 0.034; treatment lines: HR = 2.44, 95% CI = 1.31–4.56, P = 0.005). Moreover, TD independently predicted OS in univariate and multivariate analyses. Patients with TD had a 0.08-fold decreased risk of death compared with those without TD (P <0.05, shown in Figure 4).

## 4. Discussion

In the present study, we demonstrated that previous radiotherapy and patients' PS score represent predictive biomarkers for TD during camrelizumab treatment. The most common TD subtype was subclinical hypothyroidism. The occurrence of TD is a promising predictor in recurrent or metastatic ESCC patients receiving camrelizumab. TD may be related to improved PFS and OS.

The occurrence and incidence of TD varied among different immunotherapy agents. CTLA-4 inhibitors had a lower rate of TD than PD-1 or PD-L1 inhibitors [19, 20]. TD occurs in less than 5% of patients received ipilimumab. However, up to 10% of patients experienced TD when they were treated with PD-1 inhibitors. The incidence of TD was increased to about 15% in patients receiving the combination regimen [21]. Additionally, the incidence of TD is different for different ICIs, even if they are all PD-1 inhibitors [19]. In the present study, 24.4% of patients developed TD. This value is slightly different from previous reports in the literature [19]. The reason for this difference is unknown. One possible explanation is that the odds of TD is different among different PD-1 inhibitors. In the KEYNOTE-181 study [8], 10.5% of patients who received pembrolizumab monotherapy experienced grade 1–2 TD. The rate of TD increased to 15% when patients were treated with chemoimmunotherapy [22]. Camrelizumab is widely used in different lines of treatment for advanced ESCC in China. It is often associated with high rate of irAEs, especially reactive capillary hemangiomas and TD. The incidence of TD is about 19% when camrelizumab monotherapy is used [9]. When patients were treated with combination regimens, one fourth of them developed TD [23]. Another hypothesis is that more than half of patients in this study received supraclavicular radiotherapy before immunotherapy. Radiotherapy, which promotes the release of neoantigens, was associated with elevated risk of TD [24]. The increased odds of patients experiencing grade 2 or higher TD was confirmed to be related to previous supraclavicular radiotherapy for thyroid cancer [25].

Several studies demonstrated that the occurrence of TD is a promising pronostic factor in cancer patients receiving ICIs, including lung cancer [17, 18] and melanoma [26]. However, no research has investigated the association of TD and prognosis of ESCC. In our study, ESCC patients who received camrelizumab with or without chemotherapy experienced TD had better tumor response and longer OS. Moreover, multivariate analysis revealed that TD is an independent prognostic factor. Patients who experienced TD had 0.47-fold lower risk of disease progression and 0.08 times lower risk of death than those who did not. The underlying molecular mechanism behind this relationship remains unknown. The PD-1/PD-L1 signaling pathway is inhibited by camrelizumab, thereby corresponding T cells are activating. ESCC and thyroid may express many of the similar antigens. Therefore, thyroid is attacked while activating T cells attack ESCC. Angell et al. performed fine needle biopsy on the thyroid tissue of TD patients after nivolumab treatment. It was found that normal thyroid tissue was infiltrated with a large amount of cell debris and CD163 positive lymphocytes [27]. Another possible mechanism is that pre-existing chronic thyroiditis was unleashed by ICI treatment [28]. Patients with high baseline TSH concentration and positive anti-thyroid antibody (ATAb) before ICIs treatment were prone to have TD [29].

The shortcomings of our study are also obvious.. First, the nature of this study is retrospective and the sample size is relatively small. Second, PD-L1 expression was evaluated in a few patients. These factors decrease the reliability of the results. However, the patients enrolled in this study received the same ICI, not a collection of several ICIs, which can avoid bias and offset the heterogeneity. Moreover, we did not investigate the concentration of serum ATAbs before and during camrelizumab treatment. Further investigation should focus on the role of ATAbs in the prognosis of ESCC patients.

In conclusion, our study demonstrated that TD may be a new independent prognostic biomarker for PFS and OS in ESCC patients. However, further prospective studies are warranted to gain full insights into the role of TD as a promising marker during immunotherapy.

## Figures and Tables

**Table 1:** Correlations between the occurrence of TD and clinicopathological characteristics.

Variables	Number (%)	TD		P
		Absent	Present	
Gender				
Female	6 (7.3)	4 (66.7)	2 (33.3)	0.596
Male	76 (92.3)	56 (76.3)	18 (23.7)	
Age (years)				
≤ 65	53 (64.6)	38 (71.7)	15 (28.3)	0.265
> 65	29 (35.4)	24 (82.8)	5 (17.2)	
Performance status				
0	15 (18.3)	8 (53.3)	7 (46.7)	0.032
1	51 (62.2)	39 (76.5)	12 (23.5)	
2	16 (19.5)	15 (93.8)	1 (6.3)	
Smoking status				
Never	20 (24.4)	13 (65.0)	7 (35.0)	0.204
Current and former	62 (75.6)	49 (79.0)	13 (21.0)	
Alcohol status				
Never	19 (23.2)	13 (68.4)	6 (31.6)	0.405
Current and former	63 (76.8)	49 (77.8)	14 (22.2)	
Combined with chemotherapy				
No	23 (28.0)	16 (69.6)	7 (30.4)	0.426
Yes	59 (72.0)	46 (78.0)	13 (22.0)	
Treatment lines				
First	25 (30.5)	20 (80.0)	5 (20.0)	0.540
Second or more	57 (69.5)	42 (73.7)	15 (26.3)	
Tumor location				
Cervical or upper thoracic	11 (13.4)	9 (81.8)	2 (18.2)	0.819
Middle thoracic	42 (51.2)	32 (76.2)	10 (23.8)	
Lower thoracic	29 (35.4)	21 (72.4)	8 (27.6)	
Previous surgery				
No	45 (54.9)	36 (80.0)	9 (20.0)	0.307
Yes	37 (45.1)	26 (70.3)	11 (29.7)	
Previous radiotherapy				
No	37 (45.1)	33 (89.2)	4 (10.8)	0.009
Yes	45 (54.9)	29 (64.4)	16 (35.6)	
Supraclavicular radiotherapy				
No	62 (75.6)	48 (77.4)	14 (22.6)	0.502
Yes	20 (24.4)	14 (70.0)	6 (30.0)	
Stage				
III	8 (9.8)	5 (62.5)	3 (37.5)	0.363
IV	74 (90.2)	57 (77.0)	17 (23.0)	
Liver metastasis				
No	60 (73.2)	42 (70.0)	18 (30.0)	0.051
Yes	22 (26.8)	20 (90.9)	2 (9.1)	

## Data Availability

The data of this study are available from the corresponding author upon reasonable request.
